# “A living lab within a lab”: approaches and challenges for scaling digital public health in resource-constrained settings

**DOI:** 10.3389/fpubh.2023.1187069

**Published:** 2023-08-07

**Authors:** Arunima S. Mukherjee, Sundeep Sahay, Rajesh Kumar, Rashi Banta, Neha Joshi

**Affiliations:** ^1^Department of Informatics, University of Oslo, Oslo, Norway; ^2^HISP India, New Delhi, India; ^3^Health Equity Action Learnings Foundation, Chandigarh, India

**Keywords:** living lab, frugal, innovation, public health, health information systems

## Abstract

A living lab is an emerging concept, particularly in Europe, as a vehicle to develop digital innovations through a process of co-produced design and development, which takes place, physically and socially, in real-life use contexts. However, there is limited research relating to guiding our understanding of the process by which such labs are established, and digital innovations are co-created and scaled to other settings requiring similar solutions. Furthermore, beyond Europe, the concept of a living lab has not found widespread application in low- and middle-income countries (LMICs), particularly in their public health contexts. Public health systems offer the unique scaling challenge of “all or nothing”, implying that data are required from the whole population rather than isolated pilot settings. The living lab approach promises the rich potential to strengthen public systems but comes with twin interconnected challenges. First, for building appropriate digital solutions to address local public health challenges, and second, in scaling them to other public health facilities. This article investigates these twin challenges through ongoing empirical work in India and identifies three key domains of analysis, which are as follows: the first concerns the process of establishing an enabling structure of a “living lab within a lab”; the second concerns leveraging the capabilities offered by free and open-source digital technologies; and the third concerns the driving impetus to scaling through agile and co-constructed technical support.

## 1. Introduction: the challenge of scale

The application of digital technologies in public health systems continues to be rapidly accelerated in current times, particularly in the context of low- and middle-income countries (LMICs). How can the value of such digital interventions be enhanced, so that they can lead to real improvements in the access and utilization of health services, particularly, among the poor and marginalized, is the broad question this article seeks to address. This question is examined within the context of the design of digital health systems, and how they can be made more relevant to supporting health challenges in locally situated contexts. Heeks (1) argues that nearly 90% of digital health applications end up as partial or complete failures, largely contributed to by “design-reality” gaps, implying that systems designed in contexts that are geographically and culturally distant from the reality of the users are unlikely to succeed. An important question then concerns how to build design approaches to strengthen the local relevance of digital health information systems.

The question of locally relevant design is analyzed through the approach of co-production of the digital systems within a “living-lab” framework. Co-production (2) represents an approach where people, from different domains, also including members from outside the organization, work together in mutually equal ways, sharing influence, skills, and experiences to design, deliver, and monitor digital health interventions. For co-production to be effective, it arguably needs to take place within a “living lab” framework, which represents a user-centered, open-innovation ecosystem, operating in the physical setting of the user environment. The physical co-location helps to integrate concurrent processes of research and the development of innovation, by creating spaces that encourage exploration, experimentation, and evaluation of innovative ideas, within the context of real-life use cases. These processes need to consider concurrently both the global performance of the product or service being co-produced and its potential adoption by users in particular situated contexts. This brings us to consider the important question of scale.

Scaling of digital systems is a multi-faceted and multi-layered concept. This is so because the public health systems offer the unique scaling challenge of “all or nothing”, implying that planning required data is required from the whole population, rather than isolated pilot settings (3). While the lay meaning of scale implies a physical expansion, in numbers or geographical areas of use (4), recent discussions have emphasized other dimensions of scale including those related to functionality, performance, and complexity. Public health information systems represent the unique scaling challenge of “all or nothing” (5), implying that health data are needed on the whole population, else it is of limited or no value. For example, an immunization manager in a province would need to know about the vaccination status of children from all the health districts in the province and not just from a few “pilot sites”, to make meaningful interventions to achieve full immunization coverage. As a result, Braa et al. (6) argue that limited-scale systems do not get supported and are not sustainable, which constrains the scaling of the systems. Sustainability and scalability are thus inextricably interrelated.

If digital innovations to address locally relevant health challenges in a district are developed based on a living lab approach, it raises the following question of scale: “how systems and learnings developing in a particular facility of this district can be taken to other similar settings, where like problems exist, but where contexts are both similar and different.” The process of building a digital innovation through a living lab approach is both resource and time-intensive (5), making it extremely challenging for resource-constrained LMIC settings to replicate systems from scratch in multiple locations. The scaling challenge then can be expressed as: “how can learnings and systems developed in one living lab be taken to the multiplicity of locations to achieve full coverage”. This requires the scaling process to find a “pragmatic balance” (7) which can respond effectively to the paradox of the systems simultaneously having to be both globally productive and locally embedded and relevant. Unraveling this paradox theoretically and methodologically is the focus of this article, which addresses the following research question:

What are the challenges and approaches to scale living lab-generated digital innovations within multiple public health facilities in an LMIC context?

Answering this research question contributes to three domains of academic research. The first concerns ICTs for Development (ICT4D), as we highlight how technology development carried out in settings of a living lab can provide a more positive alternative than top-down approaches that typically characterize technology design approaches in LMIC contexts. The second relates to the field of public health, as professionals from this field are increasingly being exposed to digital interventions, and there is an urgent need for them to develop a more intimate understanding of challenges and approaches to making digital systems work. The third concerns the broader field of IS research, through our elaboration of the concepts of frugal innovation, scaling, and co-production, which have broader relevance than just ICT4D research.

The rest of the article is structured as follows: after providing an overview of the research problem addressed in this introduction, in the next section, we discuss relevant research, with a focus on co-production within a living lab framework and the associated challenge of scale. This is followed by a description of the methods and the case study. The case analysis and discussions follow and then brief conclusions.

### 1.1. Public health information systems in India: the need for scale

Visions of digital health innovations as vehicles for strengthening health and development processes are now dominant, particularly in LMIC settings. For example, the UN Social Development Network has stated that “Digital technologies have the capacity to foster the levels of social and economic inclusion required to achieve SDG1, poverty eradication” (unsdn.org). In 2018, the Indian Prime Minister articulated the vision of “Digital India” as being core to national development and making India the “hotspot of digital innovation across sectors … and leap-frog into the future while ensuring empowerment of every citizen” (8). However, achieving this level of digital innovation at scale (every citizen everywhere in the country) is a non-trivial challenge in terms of building a supporting ecosystem that is cost-effective, scalable, and enabling the necessary skills and knowledge (9).

We, however, acknowledge that all LMIC settings are not similar, and also some settings even in rich countries will suffer from challenges of health inequities and unequal access to healthcare services (10). India, although classified as an LMIC, has both similarities and also differences socially, culturally, and economically from other countries such as Brazil. In terms of economic growth, Brazil may be more advanced than India, but both suffer from health inequities, although for different reasons. India is overpopulated as compared to Brazil, which raises different challenges when considering scale. While many African countries produce wealth, they tend to be economically challenged and suffer from health inequities for very different reasons such as high disease burdens, poor health infrastructure, and the continued adverse impacts of the colonial legacies. The United States is a contradiction of sorts, although well-advanced in research, technology, and innovation, they have not effectively addressed health inequities. While the empirical focus is on India, we acknowledge that our findings cannot be considered applicable to all LMICs. Table 1 However, there will be some learnings that may be relevant, with appropriate incorporation of context sensitivities.

**Table 1:** Research workshops conducted during the project period.

**Workshop**	**Location**	**Participants**	**Key learnings**
No. 1	India	Institutions involved in building and studying patient-centric systems	A landscape view of patient-centric systems in India, and their strengths and weaknesses
No. 2	India	Healthcare workers and researchers	Initial design requirements for the proposed patient-centric system
No. 3	Norway	Researchers and healthcare institutions	What are the characteristics of Norwegian patient-centric systems and how are they implemented
No. 4	India	Researchers and healthcare institutions	Dissemination of results and receiving feedback on system improvements noted
No. 5	Norway	Healthcare and informatics researchers	Dissemination of results and discussions around future collaborative research
No. 6	India	Researchers and health department staff	Challenges to sustaining and scaling the system

It has been acknowledged that the Indian public health system needs to build effective health information systems (HISs) to support its engagement with the multiplicity of health challenges that they continue to experience (5). Given the size and diversity of India, it is of fundamental importance that these HISs can scale geographically and functionally, across disease domains, population groups, and geographical locations. India contributes to about 20% of the global burden of infectious diseases and 15% of global maternal deaths, has a 2% infant mortality rate (11), and only 62% of children are fully immunized (12). India ranks number one globally for tuberculosis, contributing 2.8 million new cases in 2015 (12), representing 27% of the global burden (13). India is the hotspot for non-communicable diseases, with 69 million people currently with diabetes (14), and more than one million smoking-related deaths annually (15). India was way behind its targets in achieving its MDGs, in contrast to their neighbors, such as Thailand, Sri Lanka, Bangladesh, and China.

While India has been engaged with digitization initiatives for more than two decades, they have not realized its promised potential, which can be attributed to weak systems of governance, inadequate human and institutional capacity, the limited culture of data use, and continued failures of integration efforts (5). The future also promises to bring more complexity as the nature and scale of diseases expand, more modern and complex technologies are being deployed, and new business models are being introduced through the engagement of non-state actors. Digital initiatives are expanding focus on the collection and use of personal data which heightens techno-institutional complexities (16). While the current focus on building pan-India digital systems through the National Health Authority undeniably creates the potential for providing information support to address health challenges at scale, they also come with the challenge of building systems that are simultaneously both locally relevant in situated settings, while also having the capability to be adapted to multiple other disease domains, population groups, and diverse geographical settings. To address this inter-connected challenge, our analytical focus is two-fold to understand: one, how can a “living lab” approach help in the development of HISs which are locally relevant and effective; and two, how can these systems built for a local context be scaled to other settings.

Empirically, our analysis is based on an ongoing and long-term engagement in building, implementing, and scaling an integrated patient-centric health information system for primary healthcare, within the public health system in two northern states of India. Historically, health information systems in India and also other LMICs have focussed on aggregate data for geographical areas for a state or district. These systems have been characterized by multiple vertical systems for different health programs such as Leprosy, Malaria, Maternal and Child Health, and various others. In this current project, the aim was to design and build a health information system with two crucial differences from historically existing systems. The first was to move from an aggregated system to a patient-specific system, which would record and process all transactions an individual has with the health system. The second concerns integration, which would bring data from all different health services into a unified database rather than them across multiple vertical systems. This proposed system is termed as a “patient-centric integrated HIS”, and we present an analysis of the process and challenges around their development, implementation, and scaling.

### 1.2. Co-producing and scaling locally relevant digital innovations based on a living lab framework

This section is built around four key concepts which provide the basis for the analytical framework. The first relates to ***structure***, provided by the physical and social environment of the living lab within a health facility where the system is co-produced. The second concerns the ***process*** of co-production, involving participatory and collaborative approaches, including users, developers, and researchers. The third concerns the *object* of the innovation, which in our case is the digital integrated patient-centric system, relevant for primary healthcare in an LMIC context. The fourth concerns the challenge of *scaling* the digital innovation developed through the living lab, to other settings.

The *living lab* is a relatively novel concept starting to emerge in the late 1990s, primarily promoted by the European Union for different member states. These labs initially focused on testing new technologies in home-like but constructed environments. As the concept has evolved, a precondition for a living lab today is situating the innovation work in real-world settings. A living lab has been described as an environment, a methodology, or a system (17, 18). It has also been described as a systemic innovation approach where users are seen as innovators rather than as guinea pigs, as often done in participatory design approaches (19). A common theme of living labs approaches is to promote human-centric approaches to co-produce and test digital innovations in open and collaborative real-world settings. In its requirement of a real-world setting, the living lab differs from traditional participatory design methods that often take place in classroom-like settings (20). While traditional user involvement methods focus on the designer–user interaction, the living lab seeks to involve users, producers, beneficiaries, and other relevant stakeholders (21).

Feurstein et al. (19) describe different elements characterizing a living lab including (i) participation and context; (ii) services; and (iii) methodology. Participation and context relate to the engagement of relevant stakeholders, which can unfold in single-controlled or multiple emerging settings. Services offered through a living lab include those of co-production, and adoption and implementation processes of the innovation in the living lab. A living lab by definition focuses on building something new—a product or a service—rather than based on existing products and processes. Veeckman et al. (22) analyzed the links between a living lab with its outcomes. They examined two living labs, one Flemish and the other Finnish, to understand why the former sustained and the other did not. They found the difference to be in how the value developed was shared across or not. Their study raised the important question of value, what it is, and how it is relevant or not for different stakeholders.

*Co-production* concerns the involvement of multiple stakeholders, such as designers, users, end-beneficiaries, and researchers to build sustainable partnerships (23) aimed at creating a product or service, involving people also from outside the organization (24). Another point of departure concerns the physical setting within which co-production is carried out, which is the real-life setting where digital innovation is to be used, which in our case is the specific health facility (25). While participatory processes involve varying degrees of user involvement categorized as *for, with*, and *by* (26), co-production tends to involve all three elements, although with a primary focus on the third type.

Adopting a living lab approach within a public health setting is a non-trivial challenge, given the existing system development approach that tends to be closed and top-down (27), which runs contrary to the living lab approach, emphasizing the free flow of information, knowledge, ideas, and expertise (28). Open-innovation processes in public sector organizations are arguably still in nascent stages in relation to adopting innovation models based on collaboration among citizens, entrepreneurs, and civil society (28, 29). Barriers to building more inclusive innovation include top-down and hierarchical methods of development, inadequate understanding of the public sector context, institutional barriers to collaboration, including inadequate funding (28), and the adoption of technology-deterministic approaches (30).

The ***object*** of digital innovation, in this case, is two-fold: one, concerns the digital platform itself, and two, is the particular application developed on this platform through co-production. In the first case, the digital platform used is the District Health Information Software (DHIS2, see dhis2.org) which represents a global standard for health information systems development and has been adopted in different countries for a variety of use cases, such as health program management, disease registries, logistics management, disease surveillance, and many others (20). The second case concerns the application which is co-produced on this platform, which in our case is the patient-centric integrated health information system. Building such an application represents a digital innovation for at least three reasons, which are as follows: first, traditionally primary care systems were aggregate-based (data for a facility or district), while this application represents a new form of the system based on patient-specific case-based data; second, the health system has traditionally been characterized by a multiplicity of verticalized systems, with overlaps and redundancies in the data being collected. An integrated system is novel, as it seeks to break down these vertical silos and create one common database, with a significant reduction of health workers' data load; and third, this system is based on DHIS2, an open-source digital platform, which is unique in public health systems which historically have relied on proprietary licensed software (5).

Digital platforms offer reusable and generic functions and services, which can be utilized by the innovation process to develop different components and link them as part of larger systems (31). Such platforms are flexible and potentially can be repurposed for performing a variety of tasks. Not being locked into proprietary licenses, the source code can be modified and extended to develop different required and new functionalities, including those not originally envisaged. These features make open-source digital platforms very relevant to develop digital innovations for public health systems in resource-constrained LMIC settings. As the informational needs of a public health system are forever evolving and changing, such as the addition of new data elements, indicators, organization units, and analytical features, a digital platform allows for extensions based on a modular design, without having to start from scratch. This provides relevant ingredients for the development of frugal innovations, implying the capability of doing “more with less” (32).

While LMICs have traditionally been seen as a source of “insights” for building innovations for Western countries and organizations (10), digital platforms provide the potential for change. However, also arguments such as digital technologies “has democratized innovation and almost anyone can now participate” (32; p. 3) may very well not hold in the context of LMICs because of multiple constraints of capacity, infrastructure, and prior experiences. How can these constraints be overcome represents a research and practical challenge, which this article engages with.

*Scaling* of systems represents a key challenge, which goes beyond the mere expansion of the technical artifact. The World Health Organization (33) has noted the importance of scaling to increase the impact of a technical solution on larger population groups over time. The World Bank (34) defines scaling as the process of efficiently increasing the socioeconomic impact from a small to a larger scale of coverage. Uvin (35) describes scaling as increasing the impact of grassroots organizations and their programs to move beyond being “actions on the margins” to tackling large-scale issues. Commonly understood, scaling is the process of expanding, replicating, adapting, and sustaining successful policies, programs, or projects in a geographic space and over time reaching a greater number of people. Rolland and Monteiro (7) have argued that scaling is not just physical replication but also a functional expansion, overall leading to an expansion of complexity. Puri and Sahay (36) noted that in designing and scaling a digital intervention, local needs must always be weighed against larger global needs, that encompass different communities of practice, technologies, and diverging interests and population groups. Effective scaling implies that every intervention need not start from scratch and learnings and resources built in earlier efforts can feed into new processes.

Scaling is particularly relevant to ICT4D projects, as developmental concerns are widespread, and typically resources and time are not available to start from scratch every time. Many development projects start small and even when successful, they remain rather small, especially when compared to the scale of the challenges they seek to address. In the absence of mechanisms for scaling, successful initiatives remain little more than islands of excellence in a wider economic and institutional environment (37). As a result, understanding how the expansion of the impact of such initiatives beyond the local level can be enabled has become an important issue among practitioners, donor agencies, and researchers (38). Braa et al. (6) have argued that scaling digital health interventions is a prerequisite, not a luxury, for sustainable action research, raising the challenge of *what* and *how* to scale.

Scaling is multidimensional, spanning dimensions of quantitative, functional, political, and organizational. Quantitative scaling is where a program or an organization expands its size, by increasing its membership base or its constituency or budgets (35). Spreading interventions geographically can help reach and include marginalized groups that otherwise could remain isolated and prone to continued poor health. Functional scaling is where a community-based program or a grassroots organization expands the number and the type of its activities to its operational range. This allows the system to reach more users and access a larger set of use cases (4). Political scaling refers to the extent to which participatory organizations move the use of the digital system beyond service delivery toward empowerment and changes in the structural causes of poor health. Organizational scaling is where a health organization can improve the effectiveness, efficiency, and sustainability of its activities. It can be done through different means, such as financial, networking, resource-pooling, and strengthening of informational capabilities.

While our analysis acknowledges the potential of digital platforms to technically co-produce digital innovations within a living lab framework, it notes the need for further research to understand how this potential can be effectively materialized in practice to address these multiple dimensions of scaling remains an empirical question.

## 2. Methods

### 2.1. Study context

The study is situated under two broad phases: first, a collaborative research project that involved the establishment of a living lab and to develop the digital innovation relating to the patient-centric HIS; and second, more on a project rather than a research mode, where the innovation developed were scaled to other health settings.

#### 2.1.1. Phase 1: setting up of the living lab and developing the digital innovation (2016–2019)

This started as a collaborative research project (called INTPART) involving the Department of Informatics, the University of Oslo (referred to as UiO), the Post Graduate Institute Medical and Education Research, Chandigarh, India (referred to as PGIMER), and HISP India, a local NGO and technical partner for this project. This collaboration enabled a multidisciplinary team to engage in a research and development project (from 2016 to 2019) supported by the Research Council of Norway titled “Design of Patient Centric Systems for Primary Health Care in Resource Constrained Settings”. A core aim of INTPART was to establish a living lab to enable the design and development of the digital system and support its implementation in a primary healthcare clinic in the state of Punjab.

Being a designated premier center of excellence in medical and public health research and education in India, PGIMER works closely with the state and national governments both through advisory and project implementation roles. They are assigned certain primary healthcare clinics and independently manage them with their team of doctors, field nurses, and support staff. In addition to providing care facilities to patients, they are also responsible for operating the HIS and carrying out reporting to the state and national authorities. The living lab was established in one of PGIMER's designated clinics, thus representing a “lab within a lab”. UiO is a premier institution in health information systems research and practice (see dhis2.org) and has developed the DHIS2 open-source platform used in this case. HISP India, a not-for-profit NGO, is a long-term technical partner for UiO and the state government of Punjab.

The project team based primarily at the living lab comprised 3 main groups. First, the existing medical and support team (all PGIMER staff), which is already engaged in providing health services at the health facility. Second, additional staff from PGIMER supported through INTPART, who enabled building an understanding of particular HIS informational requirements, identifying data analysis needs, and providing capacity-building support. Two senior Professors, one each from UiO and PGIMER, oversaw the research component of the project. The third group was the technical members from HISP India responsible for system design, development, and support.

#### 2.1.2. Phase 2: scaling the systems in a project mode (2021–ongoing)

This work was done outside the framework of the research project, where the systems were taken to two different locations: (i) a medical college hospital in the state of Punjab; and (ii) to 3 primary healthcare facilities in the adjoining state of Himachal Pradesh. The adaptation and implementation of the respective systems were supported by the HISP India team.

### 2.2. Data collection and analysis

#### 2.2.1. Phase 1

This involved two primary modes of engagement, including research workshops, student research, and direct primary data collection.

***Research activities*** included: (i) research workshops; (ii) research work of PhD and masters students; and (iii) everyday engagement and observations of activities in the living lab. We held six research workshops during the project, summarized in the table below.

#### 2.2.2. Research work of students

One PhD student from PGIMER and two UiO Master Students did their respective empirical work for their theses under this project. The PhD student, a medical doctor, focused on understanding the design issues of a patient-centric system from a public health perspective and its impacts on the provision of primary healthcare. Both the Masters students were from an informatics background, one focused on the process of building design requirements and the other on the challenges of designing and implementing health data standards. The PhD and Masters students had systematic processes of data collection including observations, interviews with health staff, field visits, and the study of documents and registers. Their thesis learnings contributed in different ways to the building and evolution of the patient-centric system.

#### 2.2.3. Participant observations in the living lab

This engagement served the purposes of requirements gathering and capacity strengthening of the health staff. The researchers studied the existing registers in use and understood how data were collected, recorded, and shared. We, for example, understood the interactions of health staff with patients as representing three sets of practices related to recording, tracking, and reporting data. This helped to think of the design in a modular form, representing these three sets of practices. For example, we could understand how the health ID was designed, not based on an individual but on the family, and the ID needed to also include the household location to support the health workers' outreach activities. The research team observed how the health staff interacted with the patients, the questions they asked, and the artifacts they used, all of which helped in visualizing the system. With the release of different prototypes, representing the growing understanding of the developers in how the system should look like, the health staff got increasingly involved in testing the system, providing feedback and comments, and becoming more proficient in the use of the system. This cycle represented an agile prototyping method, where design, use, and improvements were interconnected and each reinforcing the other. This helped the system design to evolve in a process that was integrated into the everyday work practices of the health staff, thus constantly adding value to practice. The researchers also gained value in gaining an intimate understanding of how a living lab worked and the workings of a patient-centric system. All the observations were documented by the researchers as notes, meeting minutes, email correspondence, test reports, capacity-building resources, and routine queries.

#### 2.2.4. Data analysis

The analysis process involved three main modes.

#### 2.2.5. Research mode

In the research mode, the Master student working on standards analyzed existing data nomenclature used in patient-centric systems, how these were aligned or not with national standards, and interventions to better synchronize them. The other student analyzed the process of requirements gathering and how they evolved and shared this information with the development team. The PhD student documented existing health status indicators before the intervention and compared them to the post-implementation status to identify the potential impacts of the digital system. Analysis emerging from these three theses contributed to informing this article in multiple ways, including the role of the living lab in enabling health innovations.

#### 2.2.6. Project mode

First, we conducted a design analysis based on meetings between the researcher team and health staff to discuss different requirements. For example, by studying the 20+ primary data registers, we could identify data duplications and eliminate duplications to build a unified database capable of generating all required output reports from the health facility. The project team conducted an infrastructure analysis as an important component of setting up the living lab, which operated in particular conditions, such as limited internet access, inconsistent electric supply, extremely hot temperatures of 45°C in summer, and limited technical expertise. The system proposed needed to be web-based, collect and integrate data from remote settings, and integrate across systems. This required making cost-effective infrastructure choices, such as for backup power supply, air humidifier, and air conditioning. Establishing a robust and resilient infrastructure to support digital development and use was an important enabling condition for the living lab.

Capacity strengthening of the health staff in using the digital system, and in thinking about how it can help add value to their everyday work was another important project-related activity. During one of the workshops, the health workers told us that their primary expectation from the system is to reduce their data collection workload. They were currently spending more than 60% of their work time on data-related work, which severely compromised their time on care provision. The design of the integrated database and the identification system was driven by this need to make their work more digital and information oriented. Furthermore, the training approach adopted was “learning by doing”, where health workers were encouraged to test the system, do data entry and generate reports independently, and ask for help from the technical team only when needed. In this way, slowly the health workers became more proficient in using the system and digital data, and slowly integrate it into their everyday work.

#### 2.2.7. Theoretical mode

The analysis represented in this article exemplifies the theoretical mode of analysis, representing a “second level of abstraction” which builds upon analytical outputs flowing from the research and project modes, which was the “first level of analysis”. For example, the research mode provided insights into the nature of digital innovations, how different technological components can be combined, and not to build something new. The infrastructure and capacity analysis helped to understand what constitutes “appropriate” technology in resource-constrained settings of primary healthcare. The theoretical mode of analysis was elaborated to understand the challenges and approaches to deal with the challenge of scale.

### 2.3. Case study

The case study is described in two parts. The first concerns the setting up of the living lab and the development of digital innovation relating to the patient-centric health information system. The second relates to the scaling process in multiple other locations.

### 2.4. Establishing a living lab within a lab

The living lab was established in a primary health clinic, which was an existing study area of the PGIMER, representing a “lab within a lab”. As a practice, most Public Health departments at medical teaching colleges in India are allocated a rural and an urban primary health center as an Intensive Field Practice Area to help medical students understand primary healthcare practices. This then provided an ideal setting to establish the living lab to develop the patient-centric system.

The health facility, like any typical primary healthcare clinic in the state, was responsible for a catchment population of about 30,000, typically including urban poor and slum dwellers, many of whom were migratory and underprivileged, dependent on the public system for routine health services. The clinic attracted an everyday patient load of about 60, and preventive, promotive, and curative services were provided by a team from PGIMER comprised of two doctors, health nurses, social workers, community volunteers, and other housekeeping staff. One software developer and a data entry operator were added through the INTPART project. Services provided included outpatient consultations, dispensing drugs, and implementing various state and national health programs, such as Maternal and Child Health, Non-Communicable Diseases, and many others. The existing information system was largely manual based on primary registers (24 in number) and the use of various forms and books for collecting, recording, and reporting data. The health staff spent more than half of their everyday time on data-related work, plus 3 to 4 full days at the end of every month for making summary reports.

The living lab was established in one room of a primary care clinic to create an arena for interaction between the PGIMER researchers, the system development, and the regular health facility staff. In this lab, the researchers also had the opportunity to observe real-time the interaction of the health staff with patients. After the room was allocated and a board established of a living lab on the door, the next important step was to equip it with appropriate infrastructure to enable the system development processes. After an initial assessment of the infrastructure, where poor internet connectivity and inconsistent power supply were identified, it was decided to go with an offline rather than online deployment of the application, supported by a high-speed server, an Uninterrupted Power Supply, and local area networking. All the desktops/laptops were connected through the local network and later, a broadband internet connection was established to enable sending automatic SMS reminders. While the local networking arrangement enabled easy local processing within the living lab, it posed challenges in scaling other health facilities in future, where web-based deployment was needed.

In terms of choices of software platforms, two free and open-source software platforms were selected, which included the DHIS2 (see dhis2.org) for data management of the outreach services provided by the nurses and the OpenMRS (see openmrs.org) for supporting the clinical patient-based work. The project plan was for the OpenMRS and DHIS2 databases to be subsequently merged to gain an overall picture of the health status of the entire 30,000 catchment population covering both outreach and clinical services.

The living lab enabled mutual learning to take place between the researchers and health staff, with the health staff understanding how the system could help them and the researcher team understanding how to reduce the data-related workload of health workers and gradually providing them with value-adding functionalities. Existing workflows were cumbersome and time-consuming, including the health worker recording data of services they provided in their field diaries, transferring that into registers, and then entering it in the computer system. The process was simplified by an automated generation of all required outputs. The researchers identified the significant need for capacity building as the health workers, who had limited experience with digital technologies, although with an intimate understanding of the data. We realized that this capacity building could not be imparted through formal training sessions, given the heavy workload of the health staff, but needed to be on the job as a part of supporting everyday work and problem-solving. A student trainee was hired to provide this on-job support.

### 2.5. Different digital innovations developed in the living lab

The co-production approach was adopted for system design, with the health staff guiding the researchers to understand the structure of the primary registers, their daily and monthly data-related routines, and practices used for culling the data from the registers to the reporting forms and then to the computer system. The clinic maintained 24 primary registers to record name-based information for each service provided, such as vaccination, TB, drugs, malaria, and antenatal check-ups, including both outreach and in-clinic services. For each service, health workers maintained the record of the person and service provided, and all follow-up encounters over time. Data from these registers were aggregated monthly to produce facility-based reports for state and national authorities. The system designers first understood how health workers identified patients in registers and what the identification system used. Rather than adding the same individual in multiple registers, there was the need identified to shift the focus from services to patients, including all individuals in the catchment population. This required a major shift in the business processes, requiring multiple digital innovations, some of which are now discussed.

#### 2.5.1. The patient identification system

To identify a patient across all registers, we understood that the unit for health service delivery was a family and not the individual. For this, the health workers used a ‘household register' to list all households/families with their home addresses and the family members in each family. The household rather than the individual then needed to be used as the unit for the identification, which also needed to indicate the location of the household, to support outreach visits of health workers. Such an understanding of the identification scheme ran contrary to the common understanding that identification should be individual-based and not traceable to the individual or household. The household-based identification would be value-adding for the health workers as they could now provide services to multiple members of a household on one visit and easily know the location of the household.

While the above identification scheme worked well for the outreach services which were based on the DHIS2 platform, it was not appropriate for the clinic-based services which were supported by the electronic medical record system based on OpenMRS. This system auto-generated a unique individual identifier at the time of a new registration through a 22-digit random number. Subsequently, a challenge was encountered in the merging of the DHIS2 and OpenMRS databases because of the two different identification schemes used, one based on the family/house and the other on the individual. As a result, patient-level data could not be transferred between the two systems. This made it a tedious task for the doctor or a health worker to search for the patient in the two applications and then match the required information. To resolve this challenge, the technical team developed a workaround. At the time of registering the patient for clinic-based care, the OpenMRS application generated a unique ID that was pasted on the patient household member page in the DHIS2 by the registration clerk, which allowed the doctor to move between the two systems by clicking on the link. However, this process of linking was cumbersome and not sustainable in the long run. For addressing this, the DHIS2 was subsequently used to develop a “lite EMR” using the DHIS2 Tracker module, which allowed the elimination of the OpenMRS application.

#### 2.5.2. Generating integrated reports

Currently, the health workers on a monthly basis prepared some 30+ facility-based reports based on source data coming from different registers. This was an extremely time-consuming and cumbersome task, taking the health workers 4–5 days a month. To automate this process, first, a unified database needed to be created including all 30,000 individuals in the catchment population; second, to include all health services data by the individuals who received them; and third, to design all required facility-based monthly reports. Taken together, this design allowed for automation where all the required monthly facility-based reports were generated by the system.

Different options were considered for building the population database. The first was to conduct a survey but this was infeasible given the costs and efforts required. The second was the option of using the national Aadhaar number (a national biometric-based identification database), but the authorities told us that this was not permitted as its use required the prior consent of citizens, and further Aadhaar did not indicate family memberships. Three, the technical team examined the option of using the public distribution database system which was family-based but was outdated. Finally, it was decided to use an incremental approach where when a person came to the dispensary, they would be asked to provide all details of their family, which was then real-time entered into the system. Gradually, through regular use, the database slowly evolved and now covers 100% of the population. With the database in place, all reports required were automated. In this process, two value-added features were added. One, new reports, not previously available, such as the patient clinical history report, were added. Two, the system could automatically generate the primary registers since all patient transactions were included in the database. This automation reduced the major pain point of the health workers in manually dealing with the 24 registers. Three, in building the population database, redundancies in data could be identified and removed, thus reducing the data load of the health workers. It is important to note that without the active and continuous engagement of the health workers, the existing pain points could not have been identified, or the solutions to address these challenges.

#### 2.5.3. Creating other value-adding functionalities

With the database and system fully operational, discussions were initiated on how the system could be made more “patient-centric.” One suggestion from the health staff was to add the SMS functionality, which was already supported by the DHIS2, to send reminders to patients to attend their appointments. These messages were sent in the local language of Hindi to enable ease of understanding and were copied to the health worker for required follow-up. As the use of this functionality took root, a Professor of Health Promotion from PGIMER suggested adding focused health promotion messages to groups of patients, such as those suffering from diabetes and hypertension. So, focused messages were designed and sent to relevant groups of patients.

At some point in this process, we met a small research group who were specializing in IVRS (Integrated Voice Response System)-based innovations, who suggested that IVRS messaging was superior to text-based SMS, because of issues of illiteracy in the populations. Furthermore, they argued that the SMS font was often too small for older adults people to read, and also because of the large numbers of SMS being received, many of the messages were left unread. These arguments led us to technically integrate the SMS and IVRS functionalities to enable sending voice messages to groups of patients. Over time, we noted a spike in the clinic attendance rates at the health facility.

### 2.6. Scaling-related challenges

As the funding cycle of the INTPART project drew to a close in 2019, several scaling challenges came to the fore. First, without continued funding, the project team hired for the living lab could not be continued. Second, the PGIMER Professor who was the driving force behind the project entered into retirement, and with this, the responsibility went to another Professor, who was not equally supportive to continue the project. Third, the two professors in the project met the state health secretary, demonstrated to him the system, and requested him to formally consider the adoption of the system for state-wide implementation. However, while the secretary was very impressed by the system and saw its value for the state, he said he did not have the authority to decide on its use, since the central government was already in the process of initiating similar systems. He said he could only consider our proposed system if it had the recommendation of the central ministry, which was not forthcoming. So, despite the technical success, the system gradually died in the health facility. However, through different circumstances, opportunities opened up to scale this system to other locations, which we describe in Phase 2.

### 2.7. Phase 2: scaling the system to other locations

In this section, we describe the process of scaling the application developed to four other health facilities, across two states, one in the same state of Punjab and three in the adjoining state of Himachal Pradesh. Interestingly, the opportunity for this scaling in both cases came through fortuitous circumstances. In the final dissemination workshop of the INTPART project, we had participation from Himachal Pradesh, who saw the system being demonstrated and invited us to introduce the system in their location. In Punjab, there was a professor in the medical college who was a colleague of the PGIMER Professor, who provided the impetus for the adoption of the system.

#### 2.7.1. Medical college in Punjab

Interestingly, similar to the earlier facility, the new medical college was another form of a “lab within a lab”. The Head was looking for a digital solution wherein details of the entire catchment population could be captured and recorded, and health services rendered appropriately through systemic data analysis. After witnessing the system demo, he called the HISP India team for a visit to his college and meet with his entire hospital team. Since the original solution was an offline solution, for the presentation, the HISP India team took a complete backup of the database, and an online instance was set up. At that time, the medical college was capturing data in paper folders and catering to a catchment population of 50,000 (urban plus rural), served through their 22 health centers, including 15 in rural villages and seven in urban areas. Medical students from the medical college were responsible to provide health services across these 22 facilities spread over 40 Sq km. One health worker, deputed through the medical college, was attached to each health center.

After the team meeting, the head was ready to adopt the system and initiated a memorandum of understanding between the college and HISP India. In the meantime, a process of requirement assessment was initiated, which took as the reference the existing patient-based system, and analyzed the additions and deletions required for the medical college. A starting point wants to study the structure and contents of the family folders by a joint team from HISP and the medical college, which yielded a detailed requirements document. This analysis identified 6 health programs that would need to be included for registering population-based services. Unlike the requirements for the original facility, in the medical college, the focus was only on outreach community services and not on clinical services. For patients coming to the facility, there was already an existing system, and it was decided not to intervene with that. This made the development far less complex, as it did not entail building integrations with the community and clinic-based data as was in the original case.

The requirement assessment document was then shared with the medical college for final approval and suggestions for change incorporated. With the final approval, HISP India started to build the system. While the original application catered to eight programs, the new systems covered a limited set of the following six programs: (i) Households; (ii) Households registration; (iii) Maternal Health; (iv) Children Under 5 years; (v) Eligible Couples; and (vi) General Individuals and Senior Citizens Screening. The first five programs were across the two systems, and the sixth was new to the medical college system. Even for the common programs, there were modifications required in the data elements, which needed to be customized. Customization could easily be done using the flexible features provided by the DHIS2. Unlike the earlier offline system, the new system was online, which was hosted on the HISP India server, enabling the development team to work continuously from remote locations. A prototype was soon created and a demo was given online, because of the travel restrictions arising from the COVID-19 pandemic.

Field implementation was severely constrained by COVID-19 as the health staff were all deployed on pandemic response responsibilities. No field training could take place as HISP India could not travel. To deal with these constraints, a local doctor was designated as the point of contact, and he was trained continuously through online means. However, delays were inherent in this situation, and only after about 8–9 months in late 2020 did the baseline data entry start at a slow pace and with dummy data. However, since the health workers were directly involved in doing the data entry work in their respective health centers, their capacity for digital work was continuously being strengthened and live data entry started in late 2021. Health workers were continuously giving feedback which was incorporated and improved versions of the revised system deployed. The health workers were motivated by seeing their requests being incorporated into the system. After baseline data were entered, including households and members, the enrolment of patients and beneficiaries was initiated in 4 health programs, and following this, follow-up data started to be captured. Custom reports and various outputs via pivot tables and dashboards were designed and made available through the system, and training was provided to the users, including a refresher training in mid-2022.

Gradually, the system was well-adopted and understood by the college staff and is now proudly owned by the medical college. In acknowledgment, the medical college has continued to extend its agreement with HISP India, which allows for continued technical support to users. Value-added outputs were gradually incorporated, such as a data status report, which allowed the college management to continuously review the progress of the use of the system and take corrective actions where required. The medical college is now considering more significant upgrades to the application, including the implementation of an Android application and procurement of the tablets are under process. HISP India has already provided the technical resources for the use of the Android application, and this would be implemented in a live setting once the tablets are procured. As of now, the system has taken deep roots, and internal capacity developed to sustain the application over time.

### 2.8. Scaling to three primary healthcare facilities in the state of Himachal Pradesh

Historically, HISP India has been an important technical support partner for digital interventions in the state since 2008, building and supporting multiple health information system applications. Given the existing trust the state already had with HISP India and that a Professor from a tertiary medical college had already seen and approved the patient-centric application in the dissemination workshop, the Department of Community Medicine invited HISP India to implement the system with due adaptations in three of their primary care facilities, one Community Health Center and two Primary Health Centers. All three facilities were study areas for the medical college, providing another lab within a lab framework. The college wanted the three facilities to implement a family folder-based approach and integrate the WHO Package of Essential Non-communicable Diseases (WHO-PEN) for primary healthcare.

Like with the earlier initiatives in Punjab, the project started with HISP India conducting a thorough requirement analysis, to document relevant information flows, and identifying the challenges in making the transition from the paper to the digital system. This requirement study was done collaboratively with users from the health facility to ensure enhanced acceptance and ownership of the system by the facility. The requirement study helped establish that the proposed system was a very good fit for the facilities. However, customizations would be required, to account for the particular hilly terrain where people lived, the kinds of occupations, and the stability of their residential arrangements. While the structure of the family folder and mapping of family members could be taken as is, the state emphasized the need to have simple rather than complicated integrated programs, which would help provide effective support for decision-making.

An assessment of hardware requirements was conducted. The project had limited funds and could only provide only one system per facility. However, there were systems available from other programs, which was deployed to this initiative. This allowed for parallel work to be conducted where some staff would be responsible for registering and creating family folders and populating them with family members' details, while others could enter program-specific data. The project outcomes demanded an online system, so that data entered at the periphery could be analyzed by the state-level program managers and view the outputs and analytical dashboards that the system was capable of generating. Given the absence of internet availability in the facilities, additional budgets were solicited and obtained for networking and server-hosting, and HISP India was made responsible for the management of the server.

Once the application was developed based on the requirements and signed off by the client, the prototype was developed and demonstrated to the medical college leaders. User credentials were then shared by the HISP team who conducted User Acceptance Testing, and feedback was received and incorporated. The beta version of the application was then released for pilot deployment, starting in one facility, and then the capacity building was conducted by the HISP India team for over 5 days. The data entered by the facility staff was monitored for 1 month and once convinced of the quality of the data, the program managers were trained on using the outputs and the analytical dashboards to support monitoring and supervision tasks. Training protocols and user manuals were developed and shared with the facility to support the future implementation and support process. User feedback was continuously received, and changes were incorporated into the application, improving and enhancing its fit with user needs.

As the system was stabilized in one facility, the hardware was procured for the remaining facilities, and the onsite training was conducted by HISP India at the other locations. This project worked very well till Oct 2022, when the nodal person for the project at the medical college was transferred to another location, which slowed progress. Project funding dried out, yet HISP India has continued to support the project because of their commitment to the cause and being located in the same state. The application continues to be used at all the facilities—sparingly in two of the facilities and very well in the other.

### 2.9. Analysis and discussions

The analysis and discussions are presented under three interconnected themes, all of which relate to the challenges and approaches to scaling a digital innovation developed through the living lab: (i) the enabling governance structure of a “living lab within a lab”; (ii) co-production processes leveraging on capabilities of free and opensource digital technologies; and (iii) the driving impetus to scaling through agile and co-produced technical support.

***The enabling governance structure of a “living lab within a lab”***: While a living lab by definition is set up within a real-use context, there is the need for a robust governance structure to ensure the lab works and delivers on its planned products and services. The living lab for the INTPART project was established within a field study area of a medical college, which had control over the working of the facility and had its staff deployed to provide routine health services. This team was then enhanced for supporting data-related processes through funding obtained for the INTPART project. However, a downside of this governance structure was that when the funding and interest from the parent lab dried out, aggravated by the retirement of the Professor driving the project, the activities in the living lab also ground to a halt. A further challenge came from the fact that the lab was set up in one primary healthcare center within a research framework and thus not well-integrated into the overall working of the official state reporting framework. When the system was shown to the health secretary to adopt it state-wide, he expressed his reluctance citing the control of these processes from the central ministry, which was planning to introduce its systems. Without such state support, it was not possible to take the system developed in one facility to the 300-odd similar facilities that existed in the state. Changes introduced in the living lab, such as the automation of the primary registers, could not be replicated in the other facilities, without a national and state-level sanction, which was not forthcoming. This experience demonstrates how governance in a public health system is organized within a multi-level structure, which makes it important to get support at all levels. The INTPART project, working in a research mode, was too focused on the processes within the living lab and ignored the linkages with the formal state system, which was necessary for scaling the system across the state.

The first case of scaling to another medical college in Punjab was also initiated within a similar framework of a lab within a lab, with the important difference that it covered 22 health facilities and not one. This larger coverage strengthened the user base through a network-based capacity. The continuing governance framework of the medical college operationalized through an institutional agreement, allowed for systems to continue to sustain. An institutional agreement ensured that the initiative was not dependent on an individual and not restricted by project-based finite funds. Similarly, in the second state, the health facilities selected for the project were within the framework of a medical college, and approvals through an agreement could be quickly initiated. However, as in the INTPART case, the continuity of the project was hampered by the transfer of the Professor championing the initiative. But given that HISP India was committed to continuing support even without funding, the project continues, although not with the same level of intensity as would have been the case with continued governance and support from the parent lab. This experience emphasizes the importance of individual champions, and their movement can potentially adversely influence the functioning and scaling of the living lab-generated innovation.

The living lab framework allows for a technical and social environment to build and scale digital innovations. As health workers “know more than what they can tell”, the physical co-location of the users and developers allows for mutual learning to take place, which otherwise would not have been possible. For example, understanding how the identification system should be designed was only made possible by the technical team observing how the health workers carried out their everyday outreach and clinical work, by studying their primary registers and engaging in discussions to understand their pain points. Similarly, health workers could gradually learn about the possibilities offered by the digital system and raise requirements, leading to a reinforcing process of mutual learning. Effective uptake of the system in one setting can provide the impetus to scale to other settings. This was seen in the dissemination workshop in the INTPART project, where the Professor from the medical college saw a good demo of the running system and was motivated to adopt it in their college facilities.

#### 2.9.1. Co-production processes leveraging on the capabilities of free and open-source digital technologies

While the living lab provided an enabling governance structure, co-production processes provided the approach to build digital innovation and provide it with relevant content. The free and open-source digital platform was critical to provide the basis on which the innovation was built within an agile framework. While the role of agile development has been noted as an important driver of digital health transformation (39), it has focused largely on the software development process. In contrast, we have used the idea of agility in a broader sense, covering the interconnected processes of design, development, and use, involving more than the stakeholders to also include researchers, health staff, technical team, and indirectly also the patients. Such agile processes, because of the proximity they entailed, could only be carried out within a living lab structure.

We saw many cases where the digital application co-produced, through the INTPART living lab, provided the vehicle for scaling, both across and within health facilities. Aiding this was the fact that all the facilities we were working in were within the uniform framework of the state public health system, which had largely similar reporting mandates, health structures (levels of reporting), and health programs against which reports had to be sent. As a result, a digital application built for one facility could technically be appropriate also for others. However, there would be changes required in specific details, such as the number of programs to include (7–8 in the first facility and 6 at the Punjab medical college) and to build a stronger focus on Non-Communicable Diseases as was the case in the health facilities at Himachal Pradesh. Such changes could be easily incorporated through the digital application, which is open source and was not locked into proprietary licenses which would not have allowed changes to the source code. The application could be deployed in offline or online modes, providing more flexibility on how technical support could be provided. The DHIS2 platform by design is flexible and easy to use, which allowed co-produced development processes to be rapidly deployed.

In addition to the geographical scaling needs, the digital platform also helped enable functional scaling. In the first case, the DHIS2 could be easily integrated with the IVRS, allowing for bulk messaging to larger groups of patients. The involvement of the Nutrition professor from PGIMER in the co-production team helped to provide value-added messaging, such as nutrition guides for diabetic patients. These different value-added features, easily deployed through the digital platform, not only helped to step up OPD attendance but also arguably contributed to improving care regimes for patients. Another technical challenge experienced related to integrating across the DHIS2 and OpenMRS systems is catering to outreach and clinical services, respectively. Given the challenge of incompatible identification systems, a lite system mimicking the clinical system of OpenMRS was quickly replicated on the DHIS2, and the problem was soon eliminated. This ability to flexibly repurpose the use of the application is a very useful capability for scaling in public health, where informational needs are dynamic and constantly evolving.

Another important enabling characteristic of digital platforms is the capability it provides to do more with less, a form of frugal innovation (32). This is an extremely important consideration for resource-constrained public health contexts in LMICs. The system also allowed for value-added features to be easily incorporated into the application, which helped go beyond mere automation, the replication of manual processes into computer form, and to make visible new information, which was previously not seen, a form of information (40). For example, through the integration of the DHIS2 and OpenMRS systems, patient clinical summary reports could be developed, which was seen as being extremely important by the doctors while providing clinical care. Furthermore, the system could be made able to generate all the primary recording registers, which helped address the biggest pain point for the health workers, given nearly half of their work time was dedicated to data work. However, these enhancements could only be made through co-production processes, where the health workers could detail their needs, and the technical team was physically and socially proximate to understanding them and converting them into useful system features.

#### 2.9.2. Driving force to scaling through agile and co-constructed technical support

The role of HISP India in providing agile technical support was crucial for the establishment of the lab (in setting up the technical environment), supporting its operations (system design, development, and support), and the scaling the application to multiple locations. Two aspects of their support process were crucial. First is related to their physical presence which allowed for agile technical support; and second, also enabled by physical presence, was the adoption of co-production principles in processes of system design and development.

Agility is crucial as the health facilities are providing healthcare, which cannot be delayed. Furthermore, they are supporting state-reporting systems that have given non-negotiable deadlines, for example, their monthly reports. So, requests coming in for technical enhancement needs to be complied with in an agile manner, both technically and institutionally, and at times, pragmatist approaches need to be adopted. For example, initially, when challenges were experienced in integrating the DHIS2 and OpenMRS data streams, an improvised solution was developed where the URL was pasted on a weblink, which the doctor could click on to view also the patient data in the other system. While this improvised solution was cumbersome, it kept the show of the care work going and bought time for the technical team to develop the clinical system on the DHIS2. New requirements coming from the health staff were rapidly incorporated into the system, leading to improvements in the system and increasing user trust in the system and technical team.

The process of co-production, representing a form of collaborative work was crucial for system development, scaling, and support. A joint participatory process to initiate the project in the requirements understanding phase was a mandatory principle adopted by HISP India. There were frequent meetings and systems demonstrations, where users would give feedback, often critical, which was always attentively listened to and responded to. This co-production process contributed to mutual learning, where the HISP India team enhanced their understanding of the domain of use, including the everyday challenges and constraints faced by the health staff, and they in turn could learn about the digital system and its potential capabilities, which also helped them to better articulate their pain points and needs.

## 3. Conclusion

Our study demonstrates how digital innovation building and scaling processes are networked (41) and involves collaboration across different organization and stakeholder groups (42). In our study, in addition to networking among the technical team, health staff, and the health system, the involvement of multidisciplinary research (informatics and public health) drawn from global, national, and local expertise was also crucial, which guided how to proceed, while learning from earlier experiences. While existing research on digital innovation has focused on Western business organizations (43), our study shows how such processes need to be adapted to particular resource-constrained public health systems. LMIC settings are typically recipients of digital systems developed in contexts culturally and geographically distant; this study highlights the strong enabling role of proximity enabled through the living lab. We acknowledge that our findings may not be relevant across all LMIC contexts, given the particularity of the Indian system. However, some general principles, such as those related to the need for multi-level governance, employing the use of co-production processes, and the need for agile techno-institutional support, could be relevant, although with necessary customizations to the local context.

This study highlights that scaling is just not a matter of replicating a technical artifact uniformly in multiple settings, but concerns a social-cultural-institutional process, where many aspects have to be considered and addressed in conjunction. The role of HISP India, built around years of existing trusting relationships with the state, is a case in point, as they were fundamental to the scaling process. The study is unique in identifying the defining role of a “lab within a lab” governance structure in the functioning of a living lab, and also the accompanying challenges. Future studies need to examine how such a governance framework can be made more robust and sustainable.

## Data availability statement

The datasets presented in this study can be found in online repositories. The names of the repository/repositories and accession number(s) can be found in the article/supplementary material.

## Author contributions

AM, SS, and RK have contributed to the conception and design of the study. RB and NJ have contributed to the empirical case. All authors contributed to manuscript revision, read, and approved the submitted version.
